# The Smartphone Peer Physical Activity Counseling (SPPAC) Program for Manual Wheelchair Users: Protocol of a Pilot Randomized Controlled Trial

**DOI:** 10.2196/resprot.7280

**Published:** 2017-04-26

**Authors:** Krista L Best, François Routhier, Shane N Sweet, Kelly P Arbour-Nicitopoulos, Jaimie F Borisoff, Luc Noreau, Kathleen A Martin Ginis

**Affiliations:** ^1^ Department of Rehabilitation Université Laval Quebec City, QC Canada; ^2^ Centre for interdisciplinary research in rehabilitation and social integration Centre intégré universitaire de santé et de services sociaux de la Capitale-Nationale Institut de réadaptation en déficience physique de Québec Quebec City, QC Canada; ^3^ Department of Kinesiology and Physical Education McGill University Montreal, QC Canada; ^4^ Centre de recherche interdisciplinaire en réadaptation du Montréal métropolitain (CRIR). Montréal, QC Canada; ^5^ Faculty of Kinesiology and Physical Education University of Toronto Toronto, ON Canada; ^6^ International Collaboration on Repair Discoveries (ICORD) Vancouver, BC Canada; ^7^ Rehabilitation Engineering Design Laboratory British Columbia Institute of Technology Burnaby, BC Canada; ^8^ Department of Kinesiology University of British Columbia (Okanagan) Kelowna, BC Canada

**Keywords:** Manual wheelchair, Physical activity, Peer training, Smartphone, Randomized controlled trial

## Abstract

**Background:**

Physical activity (PA) must be performed regularly to accrue health benefits. However, the majority of manual wheelchair users do not meet PA recommendations. Existing community-based PA programs for manual wheelchair users appear to work, but effect sizes are small and retention is low. Existing PA programs may not fully implement some psychosocial factors that are strongly linked with PA (eg, autonomy). The use of peers and mobile phone technology in the Smartphone Peer PA Counseling (SPPAC) program represents a novel approach to cultivating a PA-supportive environment for manual wheelchair users.

**Objective:**

The primary objective is to compare change in objective PA between the experimental (SPPAC) and control groups from baseline to postintervention (10 weeks) and follow-up (3 months). Changes in and relationships between subjective PA, wheelchair skills, motivation, self-efficacy (for overcoming barriers to PA for manual wheelchair use), satisfaction of psychological needs for PA, and satisfaction with PA participation will be explored (secondary outcome). Program implementation will be explored (tertiary objective).

**Methods:**

A total of 38 community-living manual wheelchair users (≥18 years) will be recruited in a randomized controlled trial (RCT). Participants in both the control and experimental groups will receive existing PA guidelines. Participants in the experimental group will also receive the SPPAC program: 14 sessions (~30 min) over a 10-week period delivered by a peer trainer using a mobile phone. PA activities will be based on individuals’ preferences and goals. Implementation of important theoretical variables will be enforced through a peer-trainer checklist. Outcomes for objective PA (primary) and subjective PA, wheelchair skills, motivation, self-efficacy, satisfaction of psychological needs, and satisfaction with participation will be collected at three time points (baseline, postintervention, follow-up). Multiple imputations will be used to treat missing data. A mixed-model ANCOVA will be conducted, controlling for covariates (primary and secondary objectives). The strength and direction of the relationships between the primary and secondary outcomes will be explored (secondary objective). Descriptive and content analysis will be used to appraise program implementation (tertiary objective).

**Results:**

Funding has been obtained from the Craig Neilsen Foundation and the Canadian Disability Participation Project, with additional funds being sought from the Canadian Institute for Health Research and Fonds de Recherche du Québec-Santé. Pilot evaluation of intervention implementation is currently underway, with enrollment anticipated to begin early 2018.

**Conclusions:**

There may be substantial benefits for the SPPAC program including limited burden on health care professionals, decreased barriers (eg. accessibility, transportation), development of peer social supports, and potential cost savings related to physical inactivity. Before conducting a large and expensive multisite RCT within a small heterogeneous population of manual wheelchair users, a pilot study affords a prudent step to establishing an adequate study protocol and implementation strategies.

**Trial Registration:**

ClinicalTrials.gov NCT02826707; https://clinicaltrials.gov/ct2/show/NCT02826707 (Archived by WebCite at http://www.webcitation.org/6pqIc14dU)

## Introduction

Despite numerous physical (eg, functioning), psychological (eg, quality of life), and social (eg, inclusion) benefits of physical activity (PA) [1], more than 55% of adults (18-74 years) and 90% of older adults (≥60 years) who use wheelchairs are not physically active enough to accrue health benefits [2,3]. The sedentary nature of sitting in a wheelchair can trigger a chain of negative physiological and psychological events that can exacerbate physical, psychosocial, and mental sequelae overtime [4]. Therefore, the health benefits of PA may be amplified for manual wheelchair users [5,6]. For instance, even moderate amounts of PA could optimize functioning and slow the spiraling effects of deconditioning that are associated with disability [7].

Compared to the general adult population, manual wheelchair users face additional barriers to PA participation, including complex health problems [8], lack of accessible spaces, transportation challenges [9], and financial stress [10]. Thus, a need for accessible and affordable PA interventions for manual wheelchair users has been documented [10]. Successful community-based programs have addressed many of these facilitators and barriers for manual wheelchair users using various behavioral approaches (eg, Workout on Wheels [10]), and individuals with spinal cord injury, who account for approximately 80% of manual wheelchair users (eg, Get in Motion [11] and home visits [12]) with variable effects on PA. Two randomized controlled trials (RCTs) of interventions that used health care professionals to prescribe PA (ie, Workout on Wheels and Get in Motion) had small effect sizes on PA [10,11]. Although cofacilitation of a home-based strength-training program by a health care professional and peer trainer had a large effect on PA (ie, strength training), findings of this study are limited by a small sample size and lack of a control group [12].

Existing community-based PA programs have implemented important variables that are known to influence PA (eg, coping, action planning, and self-efficacy). However, participant retention was less than 75% and there was little documentation of PA maintenance over time. One explanation may be that participants in these programs were generally prescribed exercise-type activities (ie, aerobic and strength-training exercises), which may have limited their perceived choice in the activities performed (ie, an important predictor of PA uptake and adherence) [13]. Arguably, improving participation in meaningful activities that require any movement may be considered PA and even small improvements in PA may have a profound impact for manual wheelchair users. Therefore, providing manual wheelchair users with choice in activities represents an important consideration.

Motivations for uptake and maintenance of PA occur through complex psychological processes. Consideration of a multitude of psychosocial variables linked with PA (eg, autonomy, motivation, self-efficacy) is required to elicit large effects on PA that result in sustainable behavior change [14-16]. Self-determination theory provides a framework for cultivating an autonomy-supportive social environment that promotes behavior change through the facilitation of the three basic psychological needs of autonomy (ie, feeling free to choose one’s own behavior), competence (ie, interacting effectively with one’s environment by mastering challenging tasks), and relatedness (ie, feeling meaningfully connected to others within one’s social group) [13-15]. Satisfaction of these basic psychological needs will lead to greater intrinsic motivation [14]. An autonomous supportive environment positively influences behavior change [17], including uptake and adherence to PA [18]. Additionally, self-efficacy (ie, an individual’s situation-specific belief in his or her capability to accomplish a given task or behavior) is one of the most salient factors in predicting uptake and maintenance of PA [16,19,20]. Although perceived competence (from self-determination theory) and self-efficacy are often used interchangeably, a clear distinction between the two concepts has been made [21]. Therefore, distinguishing between competence and self-efficacy may have important implications for better understanding the psychological mechanisms driving changes in PA behavior [21].

The tenets of social cognitive theory provide a useful theoretical lens for incorporating self-efficacy into PA interventions [19,20]. Accordingly, self-efficacy is informed by skill mastery, vicarious experience, verbal persuasion, and reinterpretation of physiological and affective symptoms [16]. Although skill mastery is the most salient of these four sources, interventions that also include vicarious experience have been shown to enhance effects on PA behavior [22]. Therefore, considering integration of theoretical factors from both self-determination and social cognitive theories represents a useful approach to tailor the specific needs of manual wheelchair users for the uptake and maintenance of PA [18].

Targeting the important theoretical factors through strategic program implementation provides one way to cultivate an autonomy-supportive environment that enhances autonomy, motivation, and self-efficacy. First, allowing individuals to choose how they participate in meaningful activities may foster a sense of perceived autonomy, which is important for enjoyment and maintenance of the behavior [13]. Second, interventions led by a peer (ie, a person who has experiential knowledge of a specific behavior and similar characteristics as the target population [23]), can enhance self-efficacy and relatedness through vicarious experiences and shared characteristics. Peers provide an effective context for modeling because they are managing comparable conditions, have experienced similar situations, and are credible [24-26]. Adults try harder and experience higher levels of learning when they learn from individuals who have perceived similarities [27]. Peers have effectively increased PA in both clinical and community settings [12,28-30], and improved manual wheelchair skills in community-living adults [31].

Finally, telephone counseling represents an accessible and affordable approach to PA counseling with promising results for increasing PA among manual wheelchair users [10,11]. Opportunely, mobile phones are becoming ubiquitous and afford greater accessibility and convenience for manual wheelchair users. The use of mobile phones for PA counseling may further target autonomy, motivations, and self-efficacy. For example, the use of mobile phones to deliver a PA intervention offers various methods of contact (eg, voice calls, video calls), which may promote personal choice and autonomy. Video calls would enable face-to-face interactions with a peer, which may further enhance self-efficacy and relatedness through vicarious experience. Additionally, social support created by using existing social apps (eg, Facebook) for communicating with peers and monitoring PA goals may provide an avenue for enhancing motivation and self-efficacy [32,33]. For example, results from a recent RCT showed online social networks comprised of like individuals had larger effects on PA than receiving promotional PA messages from a website, and at a much lower cost [33]. Although the source of motivation that arises through online social networks was unclear, the authors suggested that even minimal exposure to online social cues could have a profound influence on PA [33].

The Smartphone Peer Physical Activity Counseling (SPPAC) program will use the power of peers and technology to foster critical psychosocial constructs as a precursor to PA (ie, autonomy, motivation, self-efficacy) providing a potential solution for delivery of broad-reaching and accessible PA intervention for manual wheelchair users with minimal costs. The proposed study protocol will evaluate the efficacy of the SPPAC program for improving PA in manual wheelchair users through a pilot RCT. Based on changes in PA in the intervention group compared to the control group, the primary objective is to provide effect size estimates of the SPPAC intervention on objective PA from baseline to postintervention (10 weeks) and at follow-up (3 months postintervention). Secondary objectives include exploratory evaluation of changes and relationships in subjective PA, wheelchair skills, motivation, self-efficacy (for overcoming barriers to PA, for manual wheelchair use), satisfaction of psychological needs for PA, and satisfaction with participation in PA. Finally, implementation of the SPPAC program will be examined.

## Methods

### Trial Design

**Figure 1:**
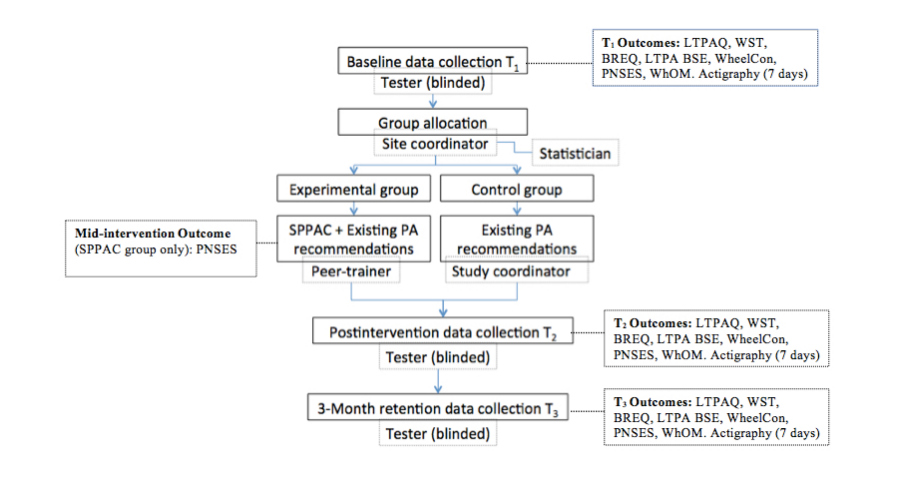
Detailed description of the Smartphone Peer Physical Activity Counseling (SPPAC) trial design and outcome assessment. HADS: Hospital Anxiety and Depression Scale, ISEL: Interpersonal Support Evaluation List, BREQ-2: Behavioral Regulation in Exercise Questionnaire-2; LTPA BSE: Leisure-Time Physical Activity Barriers Self-Efficacy scale; LTPAQ: Leisure-Time Physical Activity Questionnaire; PNSES: Psychological Need Satisfaction in Exercise Scale; WheelCon: Wheelchair Use Confidence Scale; WhOM: Wheelchair Outcome Measure; WST: Wheelchair Skills Test.

A two-site (Montreal and Quebec City, QC, Canada), single-blind, pilot RCT will be done to evaluate the efficacy of the SPPAC intervention for increasing PA in an experimental group versus a control group. Participants will be randomly assigned to the experimental group (SPPAC) or the control group (existing PA resources for manual wheelchair users) using a 1:1 allocation ratio (Figure 1). A statistician who is not part of the study team will perform randomization procedures using an undisclosed block size that is stratified by site (19 per site).

After consent and enrollment, a research assistant (RA; research professional with at least 2 years experience in clinical research) will collect baseline data and enter it into a secure database. A research coordinator will inform the statistician of enrollment via telephone or email and will obtain group assignment within 48 hours. The research coordinator will then forward the participants’ contact information to a peer trainer (experimental) and will forward PA recommendations to both groups. To reduce the risk of bias, participants will be instructed not to discuss the study with the RAs (two per site), who will be blinded to group allocation. Experimental group participants will complete 14 SPPAC sessions over 10 weeks [34]. Participants in both groups will receive usual care (ie, existing PA resources/recommendations) [35].

### Ethics

The protocol for this study was approved by the Research Ethics Boards of the Institut de réadaptation en déficience physique de Québec (IRDPQ). The study protocol was registered (NCT02826707). All study participants will provide informed consent.

### Participants

A total of 38 community-dwelling manual wheelchair users will be recruited on a volunteer basis from three large outpatient rehabilitation hospitals (IRDPQ, Jewish Rehabilitation Hospital [JRH], and Institut de réadaptation Gingras-Lindsay-de-Montréal [IRGLM]) and from two adapted fitness centers (Adaptavie, VioMax). Clinicians at the IRDPQ, JRH, and IRGLM who are part of the researchers’ respective teams will identify potential participants, whereas the fitness centers (Adaptavie, VioMax) will send information to their members by email/mail and word-of-mouth. Collaboration with Adaptavie and VioMax will also facilitate recruitment of peer trainers and may provide an avenue for sustainability of the SPPAC program in the future. Based on our past experiences in recruiting manual wheelchair users, this recruitment strategy is reasonable [1,31,36].

Participants will be between 18 and 65 years [35], live in the community, use a manual wheelchair for mobility, have used a manual wheelchair for 1 month or more, able to self-propel a manual wheelchair for at least 100 m, not be currently meeting the PA guidelines [35], able to communicate in English or French, and cognitively able to engage in the SPPAC intervention. Individuals will be excluded if they have a degenerative condition that is expected to progress quickly (eg, amyotrophic lateral sclerosis). Readiness for PA will be screened using the validated Physical Activity Readiness Questionnaire for Everyone (PAR-Q+) and the electronic Physical Activity Readiness Medical Examination (ePARmed-X+) [37,38].

### Sample Size

Based on variability data (mean, standard deviation) from Nooijen et al [39], 30 participants will be required to detect a 28-minute difference per day in PA between the experimental group and the control group (beta=.20, alpha=.05). A sample size calculation for ANCOVA in RCT designs was performed using G*power [40]. Because Arbour-Nicitopoulos et al [11] reported a 26% dropout rate in a telephone-delivered PA intervention for people living with spinal cord injury, the sample size was increased accordingly (total N=38).

### Procedure

Outcome measures will be collected at baseline (T1=prerandomization), postintervention (T2=minimum of 2 days and maximum of 10 days), and at 3-month follow-up (T3=retention) (Figure 1). Experimental bias will be minimized by having two trained RAs at each site (one will administer T1 assessments and one will administer T2 and T3 assessments).

### Intervention

**Figure 2:**
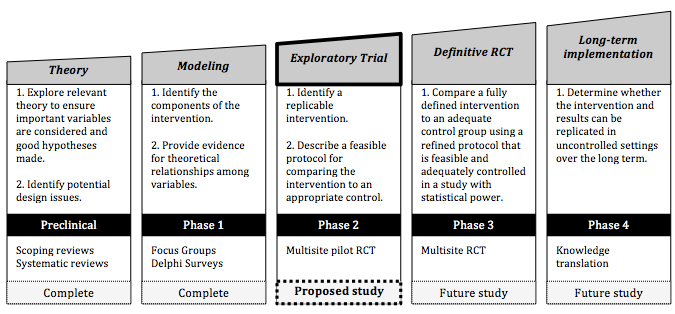
The proposed study according to the five-step process described by the Medical Research Council framework for developing complex interventions [42].

According to the Medical Research Council (MRC) framework for developing complex interventions [41], the SPPAC was established through systematic reviews and refined through focus groups and Delphi surveys with experts [34] (Figure 2). A pilot RCT is a pragmatic next step in the MRC framework to evaluate the efficacy of SPPAC for increasing PA and understanding the influence of important theoretical variables for manual wheelchair users (Figure 1).

#### Experimental Group: Smartphone Peer Physical Activity Counseling

The SPPAC program comprises 14 weekly sessions (~30 min) delivered by a peer trainer over 10 weeks. Feedback from focus groups indicated a desire for increased frequency of contact at the beginning and end of program; therefore, two sessions per week will be held during weeks 1 through 3 and week 10 [34]. Peer trainers, who are physically active manual wheelchair users with at least 5 years’ experience using a manual wheelchair, will consult a study investigator throughout program implementation as needed. A minimum of two peer trainers will receive comprehensive training in a 2-day workshop from study investigators (KB, KAN, SS) that will include education about adapted PA, PA using a manual wheelchair, manual wheelchair skills training, behavioral counseling techniques, goal setting and motivational strategies, and possible risks associated with PA among manual wheelchair users (eg, spasms, blood pressure changes, unsafe transfers).

The peer trainer will deliver the SPPAC program through voice or video calls (depending on participant preference) using a mobile phone. Video calls will be the preferred approach because face-to-face interactions may reinforce vicarious experiences (eg, the peer trainer may demonstrate how to accomplish a specific task using their wheelchair). However, preference for voice calls will be accommodated. Participants will be encouraged to visit a SPPAC private Facebook page that can be accessed through the mobile phone. The Facebook page will encourage interaction with peer trainers and other participants, creating a social network for sharing stories and pictures of PA participation, monitoring goal progression, and discussing successes and barriers. Cultivating online social networks has been shown to have a large effect on motivation for PA [33].

The initial SPPAC session will focus on rapport development and getting to know the participant. An exchange of dialog will include introductions, discussion of interests, likes and dislikes, benefits of PA, current activities performed, and PA goals and motives. The peer trainer will assist with defining PA goals for the following week and developing an action plan (eg, overcoming barriers, sources of social support [ie, SPPAC Facebook page], rewards). The peer trainer will keep a log of PA goals and action plans for each participant, and participants will also be encouraged to monitor their own goal progression (eg, writing goals and action plans). Goal setting will follow the SMART goal framework [42].

**Figure 3:**
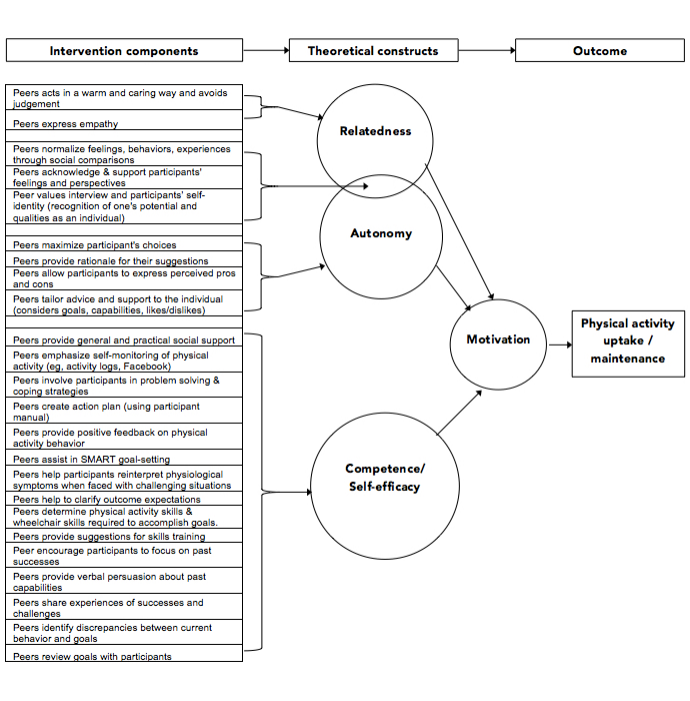
Illustration of how the components of the SPPAC intervention map onto constructs of self-determination and social cognitive theories to influence motivation and behavior change. Figure adapted from Fortier et al [18].

The remainder of the sessions will follow a standardized format of (1) 30-minute calls to review the previous week (ie, what worked/did not work, identify challenges and how they were overcome/not overcome, review/add PA goals), (2) integration of motivational strategies (eg, provide choices to modify the program to suit their individual preferences [13], brainstorm ways of making activities more enjoyable), and (3) develop action and coping plans [11,12] for the next week. The final session will follow the same format, but will also include a summary and evaluation of the SPPAC, discussion of short- and long-term goals, and relapse prevention strategies (eg, remaining part of the private SPPAC Facebook group). All sessions will be guided by a SPPAC implementation checklist, which the peer trainer will complete during each session. Figure 3 presents an illustration depicting how the components of the SPPAC intervention map onto constructs of self-determination and social cognitive theories to influence motivation and behavior change.

#### Control Group

As suggested for comparisons between experimental and control groups in pilot studies, a pragmatic research approach will be applied to determine if SPPAC is better than usual care for increasing PA [43,44]. Usual care is defined as freely available PA resources that can be accessed by everyone with no special permissions. Therefore, for minimally structuring and standardizing the control group intervention, participants will receive only a handout with existing PA guidelines and a toolkit with recommendations for PA [35]. They will not be given any instruction in how to use the information, but they will not be restricted from the uptake of PA on their own volition.

### Equipment

All participants (experimental and control group) will receive existing PA guidelines and a toolkit with recommendations for PA [35]. Participants in the experimental group will also receive a mobile phone (with a phone number, data plan, and preloaded Facebook apps). Participants may choose to use their personal mobile phone for the study if desired, but will have to download the video calling and Facebook apps and use their own data plan. On provision of the mobile phone, the tester will give instructions in how to use it. In addition, the participant manual will include step-by-step instructions in how to use the mobile phone.

### Safety

Participating in PA carries innate risks. However, the SPPAC intervention is designed to minimize risks specifically for manual wheelchair users, such as choosing appropriate activities for diagnosis, and selecting appropriate intensity and duration to minimize risk of injury. The SPPAC program incorporates strategies for safe participation, recognizing potentially unsafe situations, and seeking assistance when applicable. Participants will be asked to contact the research coordinator immediately if they experience unusual discomfort or pain. A Data and Safety Monitoring Board (statistician, researcher, health care professional, and manual wheelchair user external to the research team) will meet two times per year during the study to advise regarding safety issues [45].

### Data Collection

All outcome measures are available in English and French. Descriptive characteristics, including age, sex, marital status, education, diagnoses (ie, disability and other diseases that could contraindicate PA participation), and previous manual wheelchair experience (ie, years using a manual wheelchair, where manual wheelchair is used, hours used per day, previous wheelchair skills training received) will be collected in person at baseline by a trained RA. Potentially confounding variables, such as psychological well-being (eg, depression, anxiety) and social support, will also be collected at baseline. Depression is inversely associated with PA participation [46,47] and influences PA for manual wheelchair users [48]. The Hospital Anxiety and Depression Score (HADS), a 14-item (two 7-item subscales) self-report scale, will be used to assess depression and anxiety [49,50]. Social support is also associated with PA participation and well-being [51,52] and will be assessed using the validated 6-item Interpersonal Support Evaluation List (ISEL) [53,54].

#### Primary Outcome Measure

The primary outcome measure, PA, will be measured objectively using actigraphy with a small and lightweight accelerometry-based activity monitor (ActiGraph 3GTX) worn on the body or manual wheelchair that does not impede participation in PA [55,56]. Three-dimensional data are stored in the monitor as “activity counts” [57]. Time between sampling units (epochs) will be set at 15 seconds, allowing the greatest sensitivity for low-intensity activity [58]. In previous studies of manual wheelchair users, concurrent validity was established [58] and instrument reliability of six monitors was high (*r*^2^=.96, *P*<.001) [2].

After completion of all outcome measures at each time point, the RA will provide participants with two actigraphs (one will be positioned on the rear wheel of the manual wheelchair in a waterproof enclosure, the other will be worn on the nondominant arm). Participants will be asked to wear the Actigraph at all times over a 7-day period, except during sleep, bathing/showering, or swimming. Only data from the days in which participants wear the activity monitors for at least 13 hours per day will be included in the analyses [59].

#### Secondary Outcome Measures

Secondary outcomes reflect the proposed theoretical impacts of the SPPAC intervention. Given the dearth of evidence for manual wheelchair users in the literature, there is substantial value in understanding the relationship between PA behavior and the following variables to understand the mechanism of behavior change and to discerning a clinically important impact of the SPPAC program.

The 7-day Leisure-Time PA Questionnaire (LTPAQ) will assess self-reported PA [60]. Participants are asked to recall the frequency (number of bouts) and duration (minutes per bout) of light, moderate, and heavy intensity PA over the past 7 days. Acceptable test-retest reliability and construct validity are documented among manual wheelchair users with spinal cord injury [36,60]. Amount of PA is treated as an ordinal variable (ie, none, low, moderate, high).

The Wheelchair Skills Test-Questionnaire (WST-Q, version 4.2) will be used to assess perceived wheelchair skills capacity and performance [61]. The WST-Q is a structured assessment of 32 discrete mobility skills. WST-Q capacity is scored using a 3-point scale (0=fail; 1=pass with difficulty; 2=pass), with a maximum score of 64. A capacity score is calculated (0%-100%) reflecting the number of skills safely passed. WST-Q performance assesses how often each skill is performed on a 3-point scale (0=never; 1=sometimes; 2=always) and a performance score is calculated (0%-100%). Psychometric properties are documented for manual wheelchair users [62,63].

The Behavioral Regulation in Exercise Questionnaire 2 (BREQ-2) Revised will be used to evaluate PA motivation [64]. Four subscales measure varying degrees of exercise (or PA) regulation: external (eg, “I take part in PA because my family says I should”), introjected (eg, “I feel guilty when I do not participate in PA”), identified (eg, “It’s important to me to be physically active”), and intrinsic (eg, “I take part in PA because it is fun”) regulations. An additional subscale assesses amotivation (eg, “I think PA is a waste of time”). Each subscale contains four items except introjected regulation, which contains three items. Following the statement, “Why do you take part in PA?” participants are asked to respond to each item on a five-point Likert-type scale, ranging from 0=not at all true for me to 4=very true for me. Measurement properties of the BREQ-2 are documented [65].

The Leisure-Time PA Barrier Self-Efficacy Scale (LTPA BSE) will be used to assess self-efficacy to overcome salient barriers to PA participation (eg, when faced with transportation problems) [63]. Each of the six items is preceded with the following statement: “Assuming you were very motivated, how confident are you that you will participate in moderate to heavy leisure-time PA for at least 30 minutes on 3 days per week over the next 4 weeks even if...” Participants will be asked to rate their self-efficacy to overcome each barrier on a scale ranging from 0 to 100 (0=not confident; 50=moderately confident; and 100=completely confident). A mean score is calculated across the six items. The leisure-time PA barrier scale is reliable and valid [60,66].

The Wheelchair Use Confidence Scale-Short Form (WheelCon-SF) will be used to evaluate manual wheelchair use self-efficacy [67]. This 21-item self-report questionnaire reflects one’s current confidence using a manual wheelchair to perform various activities in varying contexts and environments. Each item is rated on a scale from 0=not confident to 10=completely confident, and a score from 0%-100% is calculated. The WheelCon-SF was derived from the original 65-item version, which has documented psychometric properties [68].

The Psychological Need Satisfaction in Exercise (PNSE) Scale will be used to assess the satisfaction of the psychological needs for PA [69]. Participants score 18 items that reflect how he/she might feel when they are physically active on a six-point Likert scale ranging from 1 (false) to 6 (true). A mean score is calculated for autonomy (6 items; “I feel free to exercise in my own way”), competence (6 items; “I feel that I am able to complete exercises that are personally challenging”), and relatedness (6 items; “I feel close to my exercise companions who appreciate how difficult exercise can be”).

The Wheelchair Outcome Measure (WhOM) will be used to assess satisfaction with participation [70]. The WhOM, a client-specific semistructured interview, allows participants to select participation goals that reflect desired outcomes of the intervention. Participants rate the “importance” of the goal (0-10) and their current “satisfaction” with performance of this activity (0-10). Goals are then integrated into the intervention. The WhOM scoring is calculated by multiplying the “importance” by “satisfaction.” Measurement properties of the WhOM have been documented [70].

#### Tertiary Outcomes

Tertiary outcomes will evaluate the implementation of the SPPAC program and ensure the SPPAC is delivered as intended. First, the peer trainer will complete a SPPAC checklist for each session, including questions about the logistics of the intervention (eg, time to complete each session, method of contact used) and a list of SPPAC components (eg, goal setting, rewards, skills training). Second, participants in the experimental group will complete a post-SPPAC questionnaire, which asks about attitude about PA, satisfaction with PA, perceived benefits and drawbacks, impact of SPPAC on PA, and changes in views of PA throughout the SPPAC intervention. Third, participants’ reported satisfaction with SPPAC will be collected with the Health Care Climate Questionnaire (HCCQ) [71]. Participants will be asked six questions about their perceived need support from their peer trainer (eg, “My peer trainer listened to how I would like to do things regarding my PA” and “I felt my peer trainer provided me with choices and options about PA”) on a 7-point Likert scale ranging from 1=strongly disagree to 7=strongly agree. High Cronbach alpha levels have been demonstrated [72,73].

### Statistical Analyses

All data analyses are conducted with SPSS version 23. Multiple imputations are used to treat missing data [74]. Data are screened for statistical outliers and assumptions for each statistical test are examined [75].

#### Primary Outcomes

Actigraphy data will be imported into Microsoft Excel in the form of activity counts, then cleaned and entered into SPSS for analysis. A mixed-model ANCOVA will be conducted, controlling for the covariates. Summary statistics (mean, SD), effect size (Cohen *d*), and significance testing (*P*) with 95% confidence intervals will be estimated.

#### Secondary Outcomes

Mixed-model ANCOVA will also be used for significance testing and effect size calculations for all secondary outcome measures. Exploratory analyses will be conducted to investigate the strength and direction of the relationships between the primary and secondary outcomes, looking for moderate-to-strong relationships [76]. If the intervention has a moderate effect on the secondary outcomes and if the secondary outcomes have moderate-to-strong relationships with primary outcome (PA), a potential mediation (exploratory) is suggested.

#### Tertiary Outcomes

Descriptive and content analysis will be used to appraise SPPAC program implementation.

## Results

This project is funded by the Craig H Neilsen Foundation and the Canadian Disability Participation Project. Proposals for additional project funds have been submitted to the Canadian Institute for Health Research (September 2016) and Fonds de Recherche du Santé Québec (December 2016). Two peer trainers have been recruited and trained and pilot evaluation of intervention implementation is currently underway. It is anticipated that enrollment for the proposed study will begin early in 2018, with final results by 2020.

## Discussion

Given the compelling evidence linking PA and quality of life, and the low rates of PA participation among manual wheelchair users, there is a clear need to find strategies to promote PA. On developing a theory-based PA program using the MRC framework, a pilot RCT is a prudent next step [41]. Establishing first effect size estimates for primary and secondary outcomes through a pilot RCT may provide justification for a subsequent efficacy RCT and variability data for future sample size calculations.

The best method to deliver PA interventions for manual wheelchair users is largely unknown. Although community-based telephone-delivered [10,11] and peer-delivered interventions [12] have shown preliminary success in manual wheelchair users, larger studies comparing modalities among this target population are required to determine how to best promote PA. The use of online social media also represents an effective solution for motivating adults without disabilities to participate in PA [33], but the influence of online social support for manual wheelchair users has yet to be evaluated. Grounded in theory, the SPPAC program of research will provide further insight about how peers, telephones, and social media can influence PA uptake and maintenance.

Behavior change is a complex and multifaceted phenomenon with multiple influencing factors. Therefore, successfully changing behavior over the long term requires multilevel interventions that consider individuals within their physical and social environments [77]. Application of two cognitive-based theories (ie, self-determination and social cognitive theories) posits that fulfilling the basic psychological needs (ie, autonomy, relatedness, and perceived control) drives behavior and that self-efficacy (synonymous to perceived control) is a strong and consistent predicator of behavior [15,16]. Therefore, cultivating an autonomy-supportive environment that enhances basic psychological needs, self-efficacy, and motivation through peer support provides a strong theoretical rationale that can effectively increase PA [18,12]. Evidence supports peer-led interventions that are grounded in social cognitive theory, both for improving PA in clinical and nonclinical populations [28] and for improving mobility outcomes for manual wheelchair users [31].

Gathering data on the important theoretical variables that are embedded within the SPPAC program will allow for exploration into mediating influences of complex psychological variables that will further our understanding about best strategies for changing PA behaviors in manual wheelchair users. The authors acknowledge the potential burden of the number of self-reported outcomes and the need for two meetings to collect objective PA data. However, it is anticipated that the self-reported outcomes can be completed in approximately 1 hour, and the tester will travel to obtain the Actigraph from the participants (or provide them with a postage-paid envelope for return to the research center) to reduce burden on participants. Future intervention studies can then integrate the most important behavior change determinants to best individualize PA programs for manual wheelchair users.

In addition to psychosocial factors, intervention dose and content represent important variables that need to be considered when promoting PA uptake and maintenance [78]. The Get in Motion program recommended up to 14 sessions of PA counseling over a 6-month period [11]. However, when asked about preferences for the SPPAC program, participants of focus groups (ie, manual wheelchair users, clinicians, and staff of community-based organizations) preferred that the SPPAC program consist of more contacts within a shorter timeframe [34]. The SPPAC program was designed accordingly [34]. In terms of optimal timing for promoting PA, a recent study among individuals with spinal cord injury suggested that the first 2 months are the most critical for successfully promoting PA [78]. However, a recent peer-led manual wheelchair training intervention showed increased participation in meaningful activities among manual wheelchair users up to 25 years after obtaining a manual wheelchair [31].

Beyond attaining knowledge of important behavioral factors influencing PA uptake and maintenance, the potential benefits of SPPAC include decreased environmental (eg, accessible facilities, transportation) and social barriers (eg, perceived stigma) to PA, limited burden on health care professionals, development of online social supports, and potential cost savings. Moreover, SPPAC has potential for application across age and diagnostic groups, a widespread geographic reach, and allows for a large trainer-to-participant ratio, all which may have time and cost efficiencies in program delivery. Future RCTs may evaluate some of the projected benefits of the SPPAC program.

Foreseen limitations of this study include the heterogeneity of the sample with regard to diagnoses. It is possible that diagnoses (as well as other sociodemographic factors, such as age) may influence the study outcomes. However, the purpose of this pilot study is to provide proof of concept of a novel intervention that may be adopted by manual wheelchair users in general. Future studies may examine diagnoses-specific interventions or consider stratification for various demographic variables. There is also a risk that participants (in both groups) may inadvertently discuss the study with other participants (eg, if they obtain health care services from the same clinic, if they are friends on Facebook) or with testers. Because the intervention is administered by phone on an individual basis, the study itself will not evoke a common meeting place for participants. Moreover, recruitment will target manual wheelchair users in remote geographic locations who may otherwise not have access to community-based programs. To reduce the risk of contamination, peer trainers will remind the participants during each session not to discuss the study with anyone outside of the intervention (including peers/friends or testers).

The SPPAC intervention aims to empower manual wheelchair users to manage their own health through implementation of autonomous supportive environments that also enhance self-efficacy through the use of a peer trainer and mobile phone technology. To our knowledge, no study has evaluated the combined potential of a mobile phone-delivered and peer-led PA intervention for manual wheelchair users. Such an approach may overcome many of the barriers to PA participation within this population, while incorporating important theoretical variables that are associated with behavior change. The SPPAC program has the potential to reach a large number of manual wheelchair users, thus may play an important role in addressing PA behavior change that could have a profound impact on health and health economics.

## Abbreviations

BREQ-2: Behavioral Regulation in Exercise Questionnaire 2
ePARmed-X+PA: electronic Physical Activity Readiness Medical Examination
HADS: Hospital Anxiety and Depression Score
HCCQ: Health Care Climate Questionnaire
IRDPQ: Institut de réadaptation en déficience physique de Québec
IRGLM: Institut de réadaptation Gingras-Lindsay-de-Montréal
ISEL: Interpersonal Support Evaluation List
JRH: Jewish Rehabilitation Hospital
MRC: Medical Research Council
PA: physical activity
PAR-Q+PA: Readiness Questionnaire for Everyone
PNSES: Psychological Need Satisfaction in Exercise Scale
RA: research assistant
RCT: randomized controlled trial
SPPAC: Smartphone Peer Physical Activity Counseling
WheelCon-SF: Wheelchair Use Confidence Scale-Short Form
WhOM: Wheelchair Outcome Measure
WST-Q: Wheelchair Skills Test-Questionnaire

## Appendix

Multimedia Appendix 1 Peer-review reports.
