# Supplementary material for: The Smartphone Peer Physical Activity Counseling (SPPAC) Program for Manual Wheelchair Users: Protocol of a Pilot Randomized Controlled Trial
Source: JMIR Res Protoc. 2017 Apr 26;6(4):e69. doi: 10.2196/resprot.7280 (PMC5425772; doi:10.2196/resprot.7280)
Supplement: Multimedia Appendix 1 [file resprot_v6i4e69_app1.pdf]

Détails du dossier - Décision

Numéro du dossier : 32092

Nom du candidat Best, Krista
Dossier 32092
Titre Active Living Lifestyles for Manual Wheelchair Users: A peer-delivered telephone approach.
Programme Formation postdoctorale pour les candidats non-résidents du Québec

Directeurs/codirecteurs de recherche ou Directeur du département

Courriel	Prenom, Nom
Luc.Noreau@rea.ulaval.ca	Noreau, Luc
Francois.Routhier@rea.ulaval.ca	Routhier, François

Acceptez-vous la bourse qui vous est offerte pour cette demande ? Oui

Etes-vous récipiendaire d'une bourse d'un autre organisme avec comités d'experts pour la durée de l'octroi offert ? Non

NOTE: Si oui, faire parvenir au FRQS une copie de l'offre de l'autre organisme.

Si vous ne désirez pas refuser l'octroi pour toute la durée offerte (ex. 1 année seulement), veuillez accepter l'octroi et inscrire les détails supplémentaires, relatifs à votre décision à la page "Informations supplémentaires".

Si vous êtes récipiendaire en 2015-2016 d'une bourse d'un autre organisme avec comité d'experts, le FRQS vous octroiera la différence dans le cas d'une bourse moindre que celle-ci. Vous devez donc accepter l'octroi offert.

NOTE: Vous aurez accès à la suite du dossier uniquement si vous acceptez l'octroi offert.

L'octroi est accepté par le candidat mais demeure conditionnel à la réception de certains documents :
Vous devez nous faire parvenir un document officiel attestant l'obtention de votre doctorat (par courriel en format PDF).
Votre directeur de formation postdoctorale doit nous confirmer par courriel le début de vos travaux de recherche (lyse.bourbonnais@frq.gouv.qc.ca).

Autorisations émises

Les montants ci-dessous sont fournis à titre informatif uniquement et peuvent différer si des modifications ont été apportées à votre dossier par le FRQS.

Année	Montant offert	Montant modifié	Conditionnel
2015/2016	30000		Oui
2016/2017	30000		Oui

Informations supplémentaires

Informations complémentaires pour la gestion de fichiers (le cas échéant)

Informations - Bourses de formation

Numéro d'assurance sociale : 125706051

DÉBUT DE LA BOURSE :

Si votre formation se poursuit au Canada, un chèque vous sera posté le 15 du mois; ce chèque couvre le mois complet. Les versements sont acheminés directement à l'adresse du laboratoire d'accueil du candidat indiqué à la page Informations financières. Veuillez indiquez le mois auquel vous aimeriez que débute votre bourse (entre mai et octobre 2015). Si votre bourse est conditionnelle, le paiement débutera lorsque la condition sera remplie. Un candidat ne peut reporter la date d'entrée en vigueur de sa bourse au-delà du 31 octobre 2015.

Mois du début de la bourse : Juillet

À quel endroit désirez-vous recevoir vos relevés d'impôt :

À l'adresse mentionnée ci-dessous.

**Adresse**	178 Kaye Street
**Ville**	Lr Sackville
**Pays**	CANADA
**Province**	Nouvelle-Écosse
**Code postal**	B4C 1N2

Mode de paiement

Si votre formation se poursuit à l'extérieur du Canada, vous aurez un chèque à tous les trois mois, c'est-à-dire quatre versements durant l'année. Nous pouvons faire parvenir vos chèques à votre laboratoire d'accueil sous forme de traites bancaires ou les poster à votre insitution financière au Québec.

Si votre formation se poursuit au Canada, choisir "Ne s'applique pas" parmi les choix offerts

Ne s'applique pas

Engagement en matière d'éthique

Identification des projets ou des programmes impliquant des sujets humains

Depuis mars 2008, Le Fonds de recherche du Québec - Santé (FRQS) exige que les détenteurs de bourses et de subventions acquièrent une formation de base en éthique de la recherche dans la mesure où leur projet ou leur programme de recherche porte sur des sujets humains.

Le terme « projet de recherche portant sur des sujets humains » inclut les projets réalisés :

Sur des sujets humains vivants;
Sur des cadavres, des embryons, des fœtus, des restes humains ou des tissus, y compris les liquides, gamètes, cellules ou du matériel génétique;
À partir de renseignements personnels contenus dans des dossiers.

Dans ce contexte et considérant cette définition, veuillez répondre à la déclaration suivante :

(En cas d'incertitude, veuillez valider cette information avec le comité d'éthique de la recherche de votre institution.)

Je déclare que mon projet ou programme de recherche, subventionné par le FRQS, porte sur des sujets humains.

Oui

Si vous avez répondu Oui à la question précédente:
Pour une bourse de formation :
Vous devez compléter la formation - niveaux 1 et 3 en cliquant sur le lien "programme de formation en éthique de la recherche". Suite à cette formation, vous devrez retourner par courriel, le certificat en format PDF au soin de la responsable du programme. La réception de ce certificat de formation est obligatoire pour que le FRQS autorise le paiement de votre deuxième année de financement.
Pour une bourse de carrière :
Vous devez compléter la formation - niveaux 1 et 3 en cliquant sur le lien "programme de formation en éthique de la recherche". Suite à cette formation, vous devrez retourner par courriel, le certificat en format PDF au soin de la responsable du programme. La réception de ce certificat de formation est obligatoire pour que le FRQS autorise le paiement de votre deuxième année de financement.
De plus, les chercheurs de carrière doivent participer comme observateur, à une réunion d'un comité d'éthique de la recherche (CER) rattaché à une université ou à un établissement du réseau de la santé et des services sociaux. Cette participation doit être réalisée dans les deux ans suivant la réponse positive du FRQS concernant leur bourse. Une lettre attestant de votre participation comme observateur, doit être émise par le CER et être expédiée au FRQS en format PDF. Cette participation doit être renouvelée lors de chaque octroi de bourse de carrière.
Pour une subvention de recherche :
Vous devez compléter (ainsi que tous les cochercheurs - excluant les collaborateurs) la formation - niveaux 1 et 3 en cliquant sur le lien "programme de formation en éthique de la recherche ". Au fur et à mesure que cette formation sera complétée, vous, ainsi que tous les cochercheurs, devrez retourner séparément par courriel, avant le début des versements, les certificats en format PDF, au soin de la responsable du programme. La réception de ces certificats de formation est obligatoire pour que le FRQS autorise le paiement de votre première année de financement.
Engagement du signataire
Le soussigné déclare et atteste ce qui suit :

J'atteste que tous les renseignements fournis sont exacts et que j'ai vérifié auprès des autorités compétentes, le cas échéant, avant de faire ma déclaration ci-dessus.

Si mon projet ou programme de recherche porte sur des sujets humains, je m'engage à fournir un certificat attestant que je me suis conformé aux exigences requises en matière d'éthique, telles que stipulées dans les conditions d'admissibilité du programme (disponibles sur le site web du FRQS), de même que chacun des cochercheurs au dossier s'il s'agit d'une subvention de recherche.
J'autorise le FRQS à échanger tous les renseignements concernant mon dossier pour fins d'études ou d'évaluation, sous la condition que les personnes ayant accès à ces renseignements en respectent le caractère confidentiel.
Je conviens que le présent engagement soit régi et interprété en vertu des lois applicables du Québec.

J'accepte : Oui

Informations financières

Laboratoire d'accueil

**Établissement**	Centre interdisciplinaire de recherche en réadaptation et intégration sociale de l'IRDPQ (CIRRIS)
**Adresse**	525 blvd Hamel
**Département**	Rehabilitation
**Au soin de**	Krista, Best
**Ville**	Quebec City
**Province**	Québec
**Pays**	CANADA
**Code postal**	G1M 2S8
**Affiliation universitaire**	Université Laval

Autorisations émises

Nombre	Année	Montant	Montant modifié	Statut	Conditionnel
1	2015/2016	30000		En cours	Oui
2	2016/2017	30000		En cours	Oui

Signature et transmission

Protection des renseignements personnels - CONSENTEMENT
Tous les renseignements de nature personnelle ou scientifique recueillis par le FRQS sont assujettis à la Loi sur l'accès aux documents des organismes publics et sur la protection des renseignements personnels (Loi sur l'accès). Le FRQS doit assurer le caractère confidentiel de ces renseignements et ne les communiquer qu'avec le consentement de la personne concernée ou conformément aux dispositions de la Loi.
Les données recueillies par le FRQS ont servi et serviront à : établir l'admissibilité des demandes; sélectionner les membres de comités d'évaluation; évaluer les demandes; assurer la gestion des programmes; procéder à des inventaires, des évaluations, des enquêtes, études ou analyses, dans le cadre de son mandat et conformément aux dispositions de la Loi sur l'accès à cet égard.
Ces activités peuvent nécessiter l'échange d'information avec les établissements de rattachement du demandeur. En acceptant le présent octroi, vous autorisez le Fonds de recherche du Québec - Santé à communiquer avec les établissements de rattachement concernés pour échanger tout renseignement relatif à la réalisation des activités énumérées précédemment.

Je m'engage à respecter les conditions émises à mon dossier pour bénéficier de l'offre d'octroi ;
Je m'engage à respecter les règles générales communes des Fonds de recherche du Québec et l'ensemble des conditions et exigences décrites sur le site web du FRQS ; Je m'engage à informer les autorités universitaires concernées, et directeur(s) de recherche s'il s'agit d'une bourse de formation. de l'offre d'octroi du FRQS ;
Je reconnais que seul le montant de l'année en cours constitue un engagement ferme de la part du FRQS. Les montants indiqués pour les années budgétaires subséquentes sont mentionnés à titre indicatif seulement. Ceux-ci peuvent être modifiés en tout temps par le conseil d'administration du FRQS, sans autre préavis, en fonction notamment des crédits qui lui sont alloués annuellement par l'Assemblée nationale du Québec, de ses priorités stratégiques et de son processus budgétaire.
J'accepte que le FRQS diffuse des données relatives à cet octroi, dont le nom du boursier ou du chercheur financé, le montant, le programme et le titre du projet, de même que le résumé vulgarisé soumis dans la demande, notamment par l'entremise de ses plateformes de communication (Web, réseaux sociaux, etc.), afin de promouvoir la recherche soutenue par des fonds publics québécois.
Je m'engage à adopter une conduite responsable en recherche et à respecter les dispositions de la Politique sur la conduite responsable en recherche du Fonds de recherche en Québec - Santé.. Advenant une allégation de manquement à la conduite responsable en recherche visant mes "activités de recherche", j'accepte que le rapport d'examen de la plainte (incluant les renseignements personnels me concernant), mené conformément aux exigences de la Politique des Fonds, soit communiqué par l'établissement aux FRQ (FRQNT, FRQS, FRQSC) afin que les Fonds puissent prendre des mesures appropriées en cas de manquement avéré. Ces mesures peuvent inclure des sanctions relatives au financement des FRQ ou à mon admissibilité à recevoir un tel financement. Si une intervention urgente était nécessaire pour prévenir ou cesser un dommage avant la fin du processus d'examen, l'établissement pourra également communiquer les informations relatives à l'allégation aux Fonds.
Je m'engage à aviser immédiatement le Fonds si je deviens non admissible à faire une demande de financement ou à détenir des fonds de recherche publics d'une agence publique de financement de la recherche au Canada ou à l'étranger, en raison d'un manquement avéré à la conduite responsable en recherche. La poursuite du soutien financer des Fonds pourra alors faire l'objet d'un examen par le comité en conduite responsable en recherche des FRQ.

Je m'engage à faire valoir la contribution financière du FRQS dans toute publication et document officiels réalisés grâce à cet octroi.

Krista Best

J'accepte: Oui

FAQ || Customer Service || Help || [X] Close Window
Best, Krista

proposalCENTRAL

Close Window Print

Proposal Information

Applicant: Best, Krista
Title Evaluation of a Smartphone-based Peer Counselling program for individuals with spinal cord injury.
Type/Competition Neilsen Postdoctoral Psychosocial Fellowships
Institution CIUSSS-CN
App # 366605

Committee: PSR 2016 FGA

Summary Statement

Text Admin Summary:
CHNF has conditionally approved funding of this proposal. Please see the reviewer comments for a listing of the strengths and weaknesses identified in your application. The following issue(s) deserve particular attention: The proposed Smartphone-based Peer Counseling (SPC) program takes an interesting but simple, cost-effective approach to the complicated health problem of physical inactivity. While the technology is not entirely novel, reviewers noted that demonstrating the feasibility and potential effect of SPC through this project could justify a larger multisite study. While the study was ambitious, the applicant is well qualified, and the research design is logical and justified. The primary and secondary mentors identified in the training plan are both highly regarded in the field and will provide complementary expertise and training. Overall, the project will support the Fellow's growth as an independent and productive researcher in the field of PSR and SCI. Reviewers had two main concerns about the training plan: 1) To strengthen the Fellowship experience, it was strongly recommended that the training plan be updated to include additional coursework on the psychological aspects of peer counseling, motivation and self-efficacy; and 2) Additional detail about the different training roles of the two mentors needs to be added. As part of the due diligence process, the applicant is asked to address these two concerns and submit an updated training plan for programmatic approval.

Reviewer Role: Primary (237823)

OVERALL IMPACT
After considering all of the review criteria, summarize the significant strengths and weaknesses of the application and state the likelihood that Neilsen Foundation funding of this project will allow this Postdoctoral Fellow to become an important contributor to the field of SCI research.

:
.The project is interesting and innovative. The senior mentor is outstanding while the primary mentor can assist with training on issues of wheelchair mobility and research training. The applicant's qualifications are very good. The environment at CIRRIS is excellent for research training.. Concerns with the complexity of the methodology proposed are expressed.The training plan should allow the candidate to get some experience in learning about psychosocial issues related to peer counseling. A course or class on the psychological aspects of counseling, motivation and self-efficacy would provide the candidate with a broader understanding of these issues.

SIGNIFICANCE
1. Does the project address an important problem or a critical barrier in the field?

2. If the Aims of the project are achieved, how would this work change or enhance current methods, technologies, treatments, services, or interventions?

3. How will the Fellow's scientific knowledge, technical capability, and/or clinical skills be improved by undertaking this project?

:
The project address an important issue, that of physical activity (PA) in SCI and proposes to use an innovative approach based on smartphone based peer counseling (SPC). There are 3 main objectives involving development, measurement and feasibility but it is unclear how these may relate to a broader goal of developing and evaluating a tele health program to modify PA behavior. The development of the smartphone approach on itself if achieved can enhance current treatments and contribute to future interventions. However, the lack of methodological clarity and its relation to this broader goal casts doubts about the significance of this study.
It is assumed that the fellow will learn to develop an innovative intervention to improve PA. However, this proposal lacks specific information about how the fellow will benefit from conducting this project during her training.

RELEVANCE
The mission of the Craig H. Neilsen Foundation is "to improve the quality of life for those living with spinal cord injury and to support scientific exploration for effective therapies and treatments leading to a cure."

The overall goal for the new Psychosocial Research (PSR) initiative is to lead to "better outcomes for people living with SCI." Emphasis is placed on research directed towards:
a) increased understanding of psychological, social and environmental determinants of health, functioning and activity participation;
b) rehabilitation and habilitation interventions to improve psychological and social functioning, including participation in work, school and other community activities;
c) improved measurements of psychological, social and environmental risk factors, protective factors, processes and outcomes; and
d) identification of critical service gaps, needed data and/or new areas of exploration, within a psychosocial or socioecological context, as defined by, or with input from, people living with SCI.

1. How is this project relevant to the mission of the Foundation?

2. How is this project relevant to the goal of the Psychosocial Research initiative?

:
The proposed project is relevant to the mission of the Craig Nielsen Foundation by proposing an innovative intervention to improve physical activity after SCI. If the project objectives are achieved it will improve our understanding of the determinants of physical activity and will contribute to our current knowledge based by proposing a new intervention to peer counseling. The application lacks specific details about the relevance of this project as part of fellowship training.

INVESTIGATOR(S)
1. Are the PI, collaborators, and other contributors well suited to the project?

2. Does the FELLOW have the potential to develop as an independent and productive researcher?

3. Are the MENTOR/SPONSOR(S)' RESEARCH QUALIFICATIONS, including research support and track record of mentoring, appropriate for the proposed Fellowship?

4. Is there evidence of a match between the research interests of the Fellow and the mentor/sponsor(s) and is there a demonstrated ability and commitment to assist in assuring the Fellow's success?

5. Do the Research and Training Plans provide the Fellow with the requisite individualized and supervised experiences that will develop his/her research skills and serve as a foundation for a productive career?

:
1. The applicant is well suited to the project and to benefit from post-doctoral training. She has a Ph.D in rehabilitation sciences from UBC and a master's degree in Kinesiology. Areas where her knowledge could be improved by this training include those of greater psychosocial relevance including her outcomes of interest: motivation, self-efficacy, and satisfaction with psychological needs, and others as listed. However, these are not discussed in relation to training.
2. The applicant has 13 publications listed of which 6 she is the primary author. This suggests a strong potential to become an independent investigator and productive researcher.
3. The primary mentor, Dr. Routhier is an Assistant Professor and appears to be well qualified from a technical viewpoint. His background, however, is in mechanical engineering, rehabilitation and geriatrics, biomedical engineering and health administration and less so in psychosocial matters. Much of his work has been in wheelchair mobility. The second mentor, Dr. Luc Noreau, is well recognized nationally and internationally and can complement these areas of mentoring based on his background and reputation.
4. There seems to be a good match between the fellow and her proposed mentors' areas of interest and expertise. Both mentors provide supportive letters of recommendation showing their commitment to this fellow's education.
5. There is no detailed information about the mentors' roles in training for this fellowship.

INNOVATION
1. Does the project challenge and seek to shift current research or clinical practice/program intervention paradigms by utilizing novel theoretical concepts, approaches or methodologies, instrumentation, or interventions?

2. And/or does this application apply concepts, approaches or methodologies, instrumentation, or interventions from another field of research to spinal cord injury?

3. And/or is a refinement, improvement, or new application of theoretical concepts, approaches or methodologies, instrumentation, or interventions proposed?

:
1. The approach proposed is not completely novel but it does utilize new technology and a new approach to peer counseling.
2. Using smartphones to encourage behavioral changes has been adopted with other chronic disease populations such as in the fields of diabetes and cardiovascular disease.
3. The project proposes the adaptation of this technology for SCI.

APPROACH
1. Is the Research Plan of high scientific quality and does it relate to the applicant's Training Plan? Are the overall strategy, methodology, and analyses well-reasoned and appropriate to accomplish the Specific Aims of the project?

2. Are preliminary data provided to support the feasibility of the project and/or are potential problems, alternative strategies, and benchmarks for success presented? Or, if the project is in the early stages of development, will the strategy establish feasibility and will particularly risky aspects be managed adequately?

3. If the project involves clinical and/or community-based research: 1) are the plans for protection of human subjects from research risks described and adequate; and 2) are the plans for recruitment of patients/participants appropriate?

:
The overall approach is briefly described and conceptualized into four distinct phases of development, measurement, feasibility leading to a RCT which is not part of this project. The approach uses a mixed method design.
The first phase (development) proposes the use of focus groups with professionals and consumers, reviews of literature. The second phase, measurement, uses a Delphi survey method to examine the proposed intervention and achieve 70% consensus. Next it will use a cross sectional design with within subjects comparisons to validate the primary outcome, actigraphy, in this study. 20 participants will be tested using their wheelchairs and examining the ability to propel themselves using both hands. Data analysis procedures are adequate for this phase although the effect of attrition or missing data is not accounted for.
The third phase, feasibility, will use a pre-posttest design to assess the feasibility of SPC investigation and other study outcomes. Study outcomes will be collected at 6 months post baseline and 3 month post intervention. The rationale for these time points is unclear plus contamination could occur between selected time points. Is 3 month follow up sufficient to demonstrate some of the selected outcomes such as increased motivation, satisfaction with participation and others. The design of this phase is not clear and lacks details in relation to the overall purpose of the study.
The discussion of participants' selection in relation to PA is also unclear. There is no mentioning of neurological classification or presence of comorbidities without much in-depth thinking about the relationship among these outcomes. Furthermore, the proposal seems overtly ambitious. For example, the applicant proposes also to explore various delivery mediums for the SPC intervention. A large number of outcomes are proposed (i.e. anxiety, depression, and social support) but their relation to PA is not clearly described. A Figure depicting the conceptual model of the proposed approach would have been most beneficial in describing the approach. Last, the sample size for phase 3 is too small given the number of variables selected. Effect sizes are discussed without specific calculations based on outcomes. Overall, the proposed approach as described is unclear.
2. There is no preliminary data relevant to the project described.
3. While the plans for recruitment of participants are described, there is no clear mentioning of using informed consents and ways of protecting confidentiality. There is a small section on safety which does not describe specific strategies to be adopted.

ENVIRONMENT
1. Is the institutional environment for the scientific development of the Fellow of high quality, and is there appropriate institutional commitment to fostering the

Fellow's training?

2. Will the research training environment provide the applicant with individualized and supervised experiences that will develop research skills needed in preparation for his/her research career?

3. Are the institutional support, physical equipment and other resources available to the investigators adequate for the project proposed?

4. Will the project benefit from unique features of the environment, subject populations, or collaborative arrangements?

:
It appears that the fellow will be placed at CIRRIS, a research center in Quebec City. Based on this information the environment will be conducive to her study and training. The institution seems committed and both mentors submitted letters of support.

NON-SCORED CRITERIA
(PLEASE NOTE THAT THE SCORE FOR THIS NON-SCORED SECTION MUST BE A "50 - NON-SCORED." PLEASE DISREGARD SCORING OPTIONS 1-9 FOR THIS SECTION ONLY.)

Please provide any important or relevant comments on each of the 4 non-scored criterion below.

1. Budget

2. Ethics/Safety

3. Other

4. Additional Comments for the Applicant

5. RESUBMISSION: When reviewing a Resubmission, the committee will evaluate the application as now presented, taking into consideration the responses to comments from the previous scientific review group and changes made to the project.

:
1. The budget seems appropriate and there are no concerns.
2. IRB approval is not discussed.
3. The training plan is missing. This is a concern since this is a resubmission and this issue was pointed out during the first submission.

Reviewer Role: Secondary (237821)

OVERALL IMPACT
After considering all of the review criteria, summarize the significant strengths and weaknesses of the application and state the likelihood that Neilsen Foundation funding of this project will allow this Postdoctoral Fellow to become an important contributor to the field of SCI research.

:
This is an ambitious application that is justified adequately and proposed by a highly qualified applicant. The smartphone based peer counseling program is moderately novel by proposing to use a peer-coach and smartphone technology to deliver a physical activity intervention. The applicant proposes a scoping review, cross sectional study to validate the primary outcome of interest for measuring physical activity, and evaluate the feasibility of the intervention in the target population. Excellent mentoring resources are available to the applicant.

SIGNIFICANCE
1. Does the project address an important problem or a critical barrier in the field?

2. If the Aims of the project are achieved, how would this work change or enhance current methods, technologies, treatments, services, or interventions?

3. How will the Fellow's scientific knowledge, technical capability, and/or clinical skills be improved by undertaking this project?

:
This project would provide modest enhancement of current methods in physical activity promotion for people with SCI. Completing the project would provide the applicant with enhanced technical capability and skills in conducting technology-enhanced behavioral change research.

RELEVANCE
The mission of the Craig H. Neilsen Foundation is "to improve the quality of life for those living with spinal cord injury and to support scientific exploration for effective therapies and treatments leading to a cure."

The overall goal for the new Psychosocial Research (PSR) initiative is to lead to "better outcomes for people living with SCI." Emphasis is placed on research directed towards:
a) increased understanding of psychological, social and environmental determinants of health, functioning and activity participation;
b) rehabilitation and habilitation interventions to improve psychological and social functioning, including participation in work, school and other community activities;
c) improved measurements of psychological, social and environmental risk factors, protective factors, processes and outcomes; and
d) identification of critical service gaps, needed data and/or new areas of exploration, within a psychosocial or socioecological context, as defined by, or with input from, people living with SCI.

1. How is this project relevant to the mission of the Foundation?

2. How is this project relevant to the goal of the Psychosocial Research initiative?

:
The project is relevant to the mission of the Foundation by seeking to enhance outcomes for people living with SCI. It is relevant to the goal of the Psychosocial Research initiative by seeking to develop rehabilitation interventions to improve the psychological and social functioning of persons with SCI by enhancing their physical and social activity.

INVESTIGATOR(S)
1. Are the PI, collaborators, and other contributors well suited to the project?

2. Does the FELLOW have the potential to develop as an independent and productive researcher?

3. Are the MENTOR/SPONSOR(S)' RESEARCH QUALIFICATIONS, including research support and track record of mentoring, appropriate for the proposed Fellowship?

4. Is there evidence of a match between the research interests of the Fellow and the mentor/sponsor(s) and is there a demonstrated ability and commitment to assist in assuring the Fellow's success?

5. Do the Research and Training Plans provide the Fellow with the requisite individualized and supervised experiences that will develop his/her research skills and serve as a foundation for a productive career?

:
The applicant and collaborators are experienced and well suited to the project; the fellow application appears to have the potential to develop as an independent and productive researcher given her training and experience. Mentors have prior experience with early career trainees. There is a good match between the interests of the fellow and mentors.

INNOVATION
1. Does the project challenge and seek to shift current research or clinical practice/program intervention paradigms by utilizing novel theoretical concepts, approaches or methodologies, instrumentation, or interventions?

2. And/or does this application apply concepts, approaches or methodologies, instrumentation, or interventions from another field of research to spinal cord injury?

3. And/or is a refinement, improvement, or new application of theoretical concepts, approaches or methodologies, instrumentation, or interventions proposed?

:
There is a large and growing body of work on physical activity promotion interventions for people with physical disabilities, including SCI. The application is moderately novel by seeking to integrate technology with peer counseling. Completion of this project will provide the applicant with experience and preliminary data to pursue larger funding sources.

APPROACH
1. Is the Research Plan of high scientific quality and does it relate to the applicant's Training Plan? Are the overall strategy, methodology, and analyses well-reasoned and appropriate to accomplish the Specific Aims of the project?

2. Are preliminary data provided to support the feasibility of the project and/or are potential problems, alternative strategies, and benchmarks for success presented? Or, if the project is in the early stages of development, will the strategy establish feasibility and will particularly risky aspects be managed adequately?

3. If the project involves clinical and/or community-based research: 1) are the plans for protection of human subjects from research risks described and adequate; and 2) are the plans for recruitment of patients/participants appropriate?

:
The research plan is detailed and organized logically. The scheduled of tasks appears to be reasonable. The rationale for the fellowship are justified adequately. The applicant provides preliminary data to justify the proposed work. Plans to recruit human subjects and protect them adequately are described.

ENVIRONMENT
1. Is the institutional environment for the scientific development of the Fellow of high quality, and is there appropriate institutional commitment to fostering the Fellow's training?

2. Will the research training environment provide the applicant with individualized and supervised experiences that will develop research skills needed in preparation for his/her research career?

3. Are the institutional support, physical equipment and other resources available to the investigators adequate for the project proposed?

4. Will the project benefit from unique features of the environment, subject populations, or collaborative arrangements?

:
The clinical research provides the subject population and other resources needed to conduct this project successfully.

NON-SCORED CRITERIA
(PLEASE NOTE THAT THE SCORE FOR THIS NON-SCORED SECTION MUST BE A "50 - NON-SCORED." PLEASE DISREGARD SCORING OPTIONS 1-9 FOR THIS SECTION ONLY.)

Please provide any important or relevant comments on each of the 4 non-scored criterion below.

1. Budget

2. Ethics/Safety

3. Other

4. Additional Comments for the Applicant

5. RESUBMISSION: When reviewing a Resubmission, the committee will evaluate the application as now presented, taking into consideration the responses to comments from the previous scientific review group and changes made to the project.

:
No response entered

©2007-2015 Altum, Inc. All rights reserved Contact Us Terms of Service Acceptable Use Policy Privacy Policy

Avis de décision - Routhier, François

Programme : Subvention Projet
Concours : 201603PJT
Numéro de la demande : 365314
Titre : Refinement and feasibility evaluation of Smartphone-delivered Peer Physical Activity Counselling (SPPAC) for manual wheelchair users.

Candidats : Routhier, François

Sweet, Shane; Best, Krista; Arbour-Nicitopoulos, Kelly; Noreau, Luc; Borisoff, Jaimie; et al

Etablissement de recherche : Université Laval

Etablissement payé : Université Laval

Résultats du concours :

Décision sur votre demande : Non-approuvée
Nombre de demandes présentées à l'étape 1 : 3819
Nombre de demandes acceptées à l'étape finale : 583

Les résultats du concours : http://www.cihr-irsc.gc.ca/f/49051.html

**Stage 1**
Décision sur votre demande : Non-approuvée
Classement combine : 74.2850
Écart-type : 30.5065

Répartition des classements des évaluateurs :

Rang centile (%)	Nombre d'évaluateurs
90%-100%	2
80%-89%	0
70%-79%	0
60%-69%	0
50%-59%	1
0%-49%	1

CIHR IRSC

Institute of Aboriginal Peoples' Health
Institute of Aging
Institute of Cancer Research
Institute of Circulatory and Respiratory Health
Institute of Gender and Health
Institute of Genetics
Institute of Health Services and Policy Research
Institute of Human Development and Child and Youth Health
Institute of Infection and Immunity
Institute of Musculoskeletal Health and Arthritis
Institute of Neurosciences, Mental Health and Addiction
Institute of Nutrition, Metabolism and Diabetes
Institute of Population and Public Health

Institut de la santé des Autochtones
Institut du vieillissement
Institut du cancer
Institut de la santé circulatoire et respiratoire
Institut de la santé des femmes et des hommes
Institut de génétique
Institut des services et des politiques de la santé
Institut du développement et de la santé des enfants et des adolescents
Institut des maladies infectieuses et immunitaires
Institut de l'appareil locomoteur et de l'arthrite
Institut des neurosciences, de la santé mentale et des toxicomanies
Institut de la nutrition, du métabolisme et du diabète
Institut de la santé publique et des populations

Le 15 juillet 2016

Docteur François Routhier
Université Laval
Département de réadaptation
Bureau 4433
Pavillon Ferdinand-Vandry
1050, avenue de la Médecine
Québec, Québec G1V 0A6

Docteur Routhier,

Les Instituts de recherche en santé du Canada (IRSC) ont évalué votre récente demande intitulée «Refinement and feasibility evaluation of Smartphone-delivered Peer Physical Activity Counselling (SPPAC) for manual wheelchair users.» présentée au concours de subventions Projet du printemps 2016. Malheureusement, votre demande n’a pas été retenue pour du financement.

Les évaluations et les résultats de votre demande sont accessibles dans RechercheNet. Si vous ne pouvez accéder aux documents, veuillez communiquer avec nous à soutien@irsc-cihr.gc.ca.

Étant donné que les IRSC n’informent pas les cocandidats de leur décision, nous vous prions de communiquer le résultat de cette demande aux personnes concernées et à leurs établissements de recherche (s’ils diffèrent du vôtre).

Pour toute question, n’hésitez pas à communiquer avec un agent de traitement du centre de contact par téléphone au 613-954-1968 ou par courriel à soutien@irsc-cihr.gc.ca.

Sincères salutations,

Martine Lafrance

Martine Lafrance, Ph.D.
Gestionnaire, Programme de subventions Projet/Direction de la Recherche libre
Portefeuille de la recherche, de l'application des connaissances et de l'éthique

432865-201603PJT-PJT-365314-97482-DLPNA

Canadian Institutes of Health Research
Room 97, 160 Elgin Street, Address locator: 4809A
Ottawa, (Ontario) K1A 0W9 Tel.: (613) 941-2672
Fax (613) 954-1800 **www.cihr-irsc.gc.ca**

Instituts de recherche en santé du Canada
Pièce 97, 160 rue Elgin, Indice de l'adresse: 4809A
Ottawa, (Ontario) K1A 0W9 Tél.: (613) 941-2672
Fax (613) 954-1800 **www.irsc-cihr.gc.ca**

Canada

**Review Type / Type d'évaluation:**	Reviewer 1 / Évaluateur 1
**Name of Applicant / Nom du chercheur:**	Routhier,François
**Application No. / Numéro de demande:**	365314
**Agency / Agence:**	CIHR/IRSC
**Competition / Concours:**	Project Grant/Subvention Projet
**Committee / Comité:**	Project Grant Competition/Concours de subventions Projet
**Title / Titre:**	Refinement and feasibility evaluation of Smartphone-delivered Peer Physical Activity Counselling (SPPAC) for manual wheelchair users.

Concept/Concept

Criterion/Critère: Quality of the Idea/Qualité de l'idée

Rating/Cote: E++

Strengths/Forces: The project is creative in that SPPAC will be refined and pilot tested for delivery by peer-coaches to manual wheelchair (MWC) users. The SPPAC integrates important psychological variables (e.g., autonomy, motivation, self-efficacy) and the latest information technologies, and was designed for physical activity uptake and adherence in MWC users. Only a limited number of MWC interventions have utilized peers to specifically target physical activity. The rationale for the project is sound because MWC users could especially benefit from physical activity, yet tend not to be active enough to experience the associated health benefits. The overall objectives of the project are well defined: 1) refine the SPPAC protocol through expert consensus from stakeholders (Delphi survey), and 2) evaluate the feasibility of SPPAC (randomized controlled trial) using appropriate feasibility indicators.

Weaknesses/Faiblesses: While employing peer-coaches to deliver the SPPAC is creative and a strength of this research project, recruiting and training these individuals may be a challenge. This is less of a concern given the research team has successfully recruited and trained MWC users to deliver interventions in their previous research.

Criterion/Critère: Importance of the Idea/Importance de l'idée

Rating/Cote: E++

Strengths/Forces: There is definitely a need for effective programs that facilitate increased physical activity by MWC users and the SPPAC has the potential to be such a program. Demonstrating the feasibility of the SPPAC program will support future research (MRC framework phases 3 and 4), and eventually the SPPAC program may lead to decreased co-morbidities among MWC users through increased physical activity participation.

Weaknesses/Faiblesses: While this project will indicate if with appropriate training, peer-coaches can effectively offer the SPPAC program to MWC users, what will remain a concern is whether the SPPAC program delivered by peer-coaches is possible to offer on a larger scale. The SPPAC program may prove to be effective, but the delivery of this program may be more problematic.

Feasibility/Faisabilité

Criterion/Critère: Approach/Approche

Rating/Cote: E+

Strengths/Forces: Employing the MRC framework for developing and evaluating the SPPAC program is a strength of the proposed research. Another strength is that the content of the SPPAC program seems to have a strong theoretical basis, as well as being guided by previous research. The approaches to complete phases 2 and 3 of the MRC framework are sound. In phase 2, a Delphi procedure will follow an iterative process by a panel of experts to achieve consensus on the content and delivery of SPPAC. A multi-site (Quebec, Montreal, Vancouver) randomized-controlled trial will be used in phase 3 to assess the feasibility of SPPAC. The number and types of measures being assessed in the RCT (i.e., indicators of process, resources, management, and treatment, as well as effect size estimates for objective physical activity, self-report physical activity, MWC mobility, physical activity motivation, physical activity and MWC use self-efficacy, autonomy support,

satisfaction of the psychological needs for physical activity, and satisfaction with participation in physical activity) are a strength of this study. The timelines and related deliverables of the project are realistic. The potential challenges and some possible mitigation strategies are discussed.

Weaknesses/Faiblesses: Given the focus groups have already been conducted in phase 1 of the MRC framework and the SPPAC program is to be refined in this research project, further information about the SPPAC program should be provided. That is, describe what is being refined. If the focus group study is still being completed, this represents a definite weakness. In addition, more detail regarding the training provided to the peer-coaches in the 2-day workshop should be included.

Criterion/Critère: Expertise, Experience and Resources/Expertise, expérience et ressources

Rating/Cote: O+

Strengths/Forces: The project is being conducted by a strong research team led by Dr Routhier. These researchers certainly have the experience and expertise to complete the proposed project and deliver the proposed outputs. Given the size and diversity of the team, the commitments being proposed for each team member generally seem appropriate. Moreover, the team appears to have the resources and infrastructure necessary to successfully complete this research.

Weaknesses/Faiblesses: Including another team member associated with the Vancouver site might be worthwhile.

**Review Type / Type d'évaluation:**	Reviewer 2 / Évaluateur 2
**Name of Applicant / Nom du chercheur:**	Routhier,François
**Application No. / Numéro de demande:**	365314
**Agency / Agence:**	CIHR/IRSC
**Competition / Concours:**	Project Grant/Subvention Projet
**Committee / Comité:**	Project Grant Competition/Concours de subventions Projet
**Title / Titre:**	Refinement and feasibility evaluation of Smartphone-delivered Peer Physical Activity Counselling (SPPAC) for manual wheelchair users.

Concept/Concept

Criterion/Critère: Quality of the Idea/Qualité de l'idée

Rating/Cote: E

Strengths/Forces: This study is novel in that it studies the use of smartphone-delivered peer physical activity counselling (SPPAC) to increase the physical activity participation of persons using manual wheelchairs based on theories of behaviour from Self-Determination Theory and Social Cognitive Theories. Apparently this particular intervention has not been studied before. Phase 1 of the study will use expert opinion to refine an existing SPPAC program and phase 2 will conduct an RCT to explore the feasibility of conducting a larger RCT & to provide preliminary evidence of the effectiveness of SPPAC.

Weaknesses/Faiblesses: This seems to be a large & expensive study to simply show the feasibility of doing a much larger study. I don’t understand why a comparison group is needed to show that the SPPAC program can be delivered to manual wheelchair users & that various outcomes can be measured. I recognize the MRC recommendation of a Phase II or Exploratory trial but I am not convinced that this study captures the benefits of an Exploratory trial, e.g., varying different components of the intervention to see what effect each has on the intervention as a whole. JUN 18/16 - On re-reading the MRC Framework document on the value of Exploratory Trials, I can see that there is merit in testing the feasibility of conducting a larger RCT & that means including a comparison group

Criterion/Critère: Importance of the Idea/Importance de l'idée

Rating/Cote: E+

Strengths/Forces: A sizeable number of people use wheelchairs & the majority do not exercise regularly thus placing them at increased risk of chronic disease. Much is known about the complex variables influencing PA uptake & adherence, e.g., peer support & telephone-delivered interventions, but they have not been incorporated into PA strategies. A pilot focus group identified numerous pros of SPPAC.

This study will refine an existing SPPAC prototype intervention using focus groups & a Delphi survey considering barriers, facilitators, appropriate PA adaptations, methods of delivery, and theoretical variables. Then it will conduct an RCT with manual wheelchair users comparing those receiving the refined SPPAC intervention with a control group not receiving the intervention. The goal is to demonstrate the feasibility of the SPPAC and provide initial evidence of its effectiveness as justification to conduct larger scale studies of SPPAC. It has the potential to be a state-of-the-art program that could improve the health of manual wheelchair users & significantly reduce their cost of care.

Weaknesses/Faiblesses: I had trouble understanding the section on feasibility indicators.

Feasibility/Faisabilité

Criterion/Critère: Approach/Approche

Rating/Cote: E+

Strengths/Forces: The numbers of participants in the RCT, the selection & exclusion criteria and the timing to introduce the SPPAC of at least 1 year after starting to use a manual wheelchair all appear appropriate. The selection &

training of the peer coaches appears appropriate. Attention to the safety of the participants is addressed. Sample size justification is based on a similar study of patients with SCI and takes account of the use of 3 study sites.

Weaknesses/Faiblesses: It was not clear how the Delphi results, expert feedback and answers to open-ended questions would be integrated to create the version of the SPPAC used in the study. I am not convinced that the addition of a control group in this study adds very much. It is not essential to address the feasibility questions. JUN 17/16 However...it is an important step in preparation for a larger RCT as described by the MRC recommendations.

Criterion/Critère: Expertise, Experience and Resources/Expertise, expérience et ressources

Rating/Cote: O

Strengths/Forces: Strong team with relevant expertise:

•Successful collaboration on >10 national grants, several related to exercise

•Several millions of grants including several studies of persons with disabilities or using wheelchairs

•Includes a rehabilitation engineer, 3 exercise psychologists, KT experts, kinesiologist with experience in intervention evaluation including peer-led interventions and PA, expertise in intervention development and PA measurement, expert in technology development, long-term wheelchair user

•Collaboration with users of manual wheelchairs

Statistician

Weaknesses/Faiblesses: I can’t tell if the technical expertise includes expertise in developing software for smartphones.

**Review Type / Type d'évaluation:**	Reviewer 3 / Évaluateur 3
**Name of Applicant / Nom du chercheur:**	Routhier,François
**Application No. / Numéro de demande:**	365314
**Agency / Agence:**	CIHR/IRSC
**Competition / Concours:**	Project Grant/Subvention Projet
**Committee / Comité:**	Project Grant Competition/Concours de subventions Projet
**Title / Titre:**	Refinement and feasibility evaluation of Smartphone-delivered Peer Physical Activity Counselling (SPPAC) for manual wheelchair users.

Concept/Concept

Criterion/Critère: Quality of the Idea/Qualité de l'idée

Rating/Cote: O

Strengths/Forces: The goal of the proposed study is to refine a Smartphone-delivered Peer Physical Activity Counselling (SPPAC) intervention to evaluate its feasibility prior to launching a larger trial.

The objectives of the proposed research are well defined.

The use of peer counseling, particularly among special populations, and examining the feasibility of using technology to deliver peer counseling warrants exploration.

Weaknesses/Faiblesses: -

Criterion/Critère: Importance of the Idea/Importance de l'idée

Rating/Cote: O

Strengths/Forces: Physical inactivity-related chronic disease is a concern among the spinal cord injury population. There is a demonstrated need for interventions targeting manual wheelchair users (MWC) and although programs have been developed, adherence rates are low. Peer-led interventions have been shown to be effective for increasing self-efficacy but there is limited research focusing on the effect of peers influencing PA among MWC users. Given the high rates of inactivity and increased risk of developing chronic disease, the proposed research has the potential to improve health outcomes among MWC users. Phone-delivered interventions are a preferred method of intervention delivery among the SCI population and thus exporing the use of mobile phone technologies may enhance delivery of interventions by capitalizing on other features of smart phones such as video and text-reminders, in additional to traditional phone calls.

Weaknesses/Faiblesses: -

Feasibility/Faisabilité

Criterion/Critère: Approach/Approche

Rating/Cote: E++

Strengths/Forces: The Medical Research Council framework for developing and evaluating complex interventions is being used to guide the research.

The approach involves using the Delphi technique to refine the SPPAC protocol. Components to be presented to experts include delivery methods, intervention duration, frequency of sessions and contact as well as content.

A 3-site RCT will be used to assess the feasibility of the SPPAC. This is a pragmatic trial, which is suitable for the target population. The feasibility indicators are comprehensive.

Investigational outcomes are also comprehensive and include accelerometry to measure PA, LTPA, wheelchair skills, PA

motivation, perceived autonomy support, satisfaction of psychological needs, barriers to PA and self-efficacy for using a MWC. A semi-structured interview will explore satisfaction with PA participation.

Challenges and mitigating strategies have been highlighted

Weaknesses/Faiblesses: Protocol refinement – the analysis indicates audio-recordings will be transcribed, however it is unclear when recordings would take place as it appears all the questions to experts will occur via email.

Feasibility Study – it is unclear what constitutes success of the intervention. How will fidelity of the intervention be evaluated?

Criterion/Critère: Expertise, Experience and Resources/Expertise, expérience et ressources

Rating/Cote: O

Strengths/Forces: The NPA has expertise in MWC mobility, intervention development and PA measurement. Expertise of team members include exercise psychology, occupational therapy, kinesiology and experience working with the SCI population, notably Martin-Ginis. This is a strong team with the expertise to conduct the proposed research.

Weaknesses/Faiblesses: -

**Review Type / Type d'évaluation:**	Reviewer 4 / Évaluateur 4
**Name of Applicant / Nom du chercheur:**	Routhier,François
**Application No. / Numéro de demande:**	365314
**Agency / Agence:**	CIHR/IRSC
**Competition / Concours:**	Project Grant/Subvention Projet
**Committee / Comité:**	Project Grant Competition/Concours de subventions Projet
**Title / Titre:**	Refinement and feasibility evaluation of Smartphone-delivered Peer Physical Activity Counselling (SPPAC) for manual wheelchair users.

Concept/Concept

Criterion/Critère: Quality of the Idea/Qualité de l'idée

Rating/Cote: E++

Strengths/Forces: Although research on technology driven counselling is increasing, the proposed study has a novel approach in focusing on the development of a counselling tool for manual wheelchair users. Work has occurred for gathering evidence to inform the development of the tool. The rationale seems sound and the project goals have been well defined.

Weaknesses/Faiblesses: Assumptions about the delivery mechanism are based somewhat on slightly different interventions. Although understandable about the potential reach and cost effectiveness of using mobile devices as the primary delivery mechanism in this intervention, there are some concerns if there will be the potential to preclude some adults who are not technologically savvy or do have use have a device (e.g. receptivity of technology with age) given in the justification of the study, 8% of older MWC users are not active. Has there been any consideration of a mixed mode of delivery? Peer counselling is an interesting approach, however, would this be feasible on a larger scale?

Criterion/Critère: Importance of the Idea/Importance de l'idée

Rating/Cote: E+

Strengths/Forces: The research is an interesting and important topic with the potential impact of positively increasing activity rates among manual wheelchair users. There is a relative lack of research in the area of technology integration with counselling among this population, which this study aims to address. The counselling intervention has the potential to impact behaviour of this population at relatively low cost, and administration seems feasible.

Weaknesses/Faiblesses: Some considerations with approach can be considered in the development of the RCT. RCT specifications are determine to a certain extent on the outcome of the consensus phase of the project.

Feasibility/Faisabilité

Criterion/Critère: Approach/Approche

Rating/Cote: E+

Strengths/Forces: Strengths include theoretically based constructs, use of objective measures, barrier and self efficacy scales associated with wheelchair use and competence. The approach for the RCT seems appropriate although it will be modified based on the consensus phase. Timelines seem appropriate for the study. Challenges that have been identified seem to be addressed in the proposal in an acceptable way, including honoraria to reduce drop outs, and partnerships to aid in recruitment.

Weaknesses/Faiblesses: Are sex, and particularly age, considered for the selection process? Geographical considerations with respect to wheelchair use will need to be considered. Why was a leisure time physical activity measure selected for self-reported frequency as opposed to a overall activity measure which could incorporate transportation and work related activity? It was unclear from the description how the study will be refined based on the results of the first phase of the study. Estimates of the dropout rate seem low, however, estimates are expected to be similar to the telephone methodology used with SCI participants in an earlier study.

**Review Type / Type d'évaluation:**	Reviewer 4 / Évaluateur 4
**Name of Applicant / Nom du chercheur:**	Routhier,François
**Application No. / Numéro de demande:**	365314
**Agency / Agence:**	CIHR/IRSC
**Competition / Concours:**	Project Grant/Subvention Projet
**Committee / Comité:**	Project Grant Competition/Concours de subventions Projet
**Title / Titre:**	Refinement and feasibility evaluation of Smartphone-delivered Peer Physical Activity Counselling (SPPAC) for manual wheelchair users.

Criterion/Critère: Expertise, Experience and Resources/Expertise, expérience et ressources

Rating/Cote: O

Strengths/Forces: The team appear to be varied and well positioned to ensure that the project is successfully completed. The level of commitment from the principal investigator and co-investigators seem appropriate based on the approach and budget justification.

Weaknesses/Faiblesses: No identifiable weaknesses.

NOTE

Your application was assessed by the Stage 1 reviewers. Considering the rating and ranking of your application relative to the other applications in the competition, your application did not move to the Final Assessment Stage (FAS). As a result, your application was not discussed by the Final Assessment Stage committee and no Scientific Officer notes were generated. The reviewers' reports from the first stage are available on ResearchNet.

Please refer to the *Notice of Decision* for more information regarding the ranking of your application.

For information regarding the Project Grant Program peer review process, please refer to the *Peer Review Manual - Project* (http://www.cihr-irsc.gc.ca/e/49564.html).

Votre demande a été évaluée par les évaluateurs de l'Étape 1. Compte tenu de sa cote et de son rang par rapport aux autres demandes dans le concours, votre demande n'est pas passée à l'étape d'évaluation finale (EEF). Par conséquent, votre demande n'a pas fait l'objet d'une discussion par le comité de l'étape d'évaluation finale. Il n'y a donc pas de notes de l'agent scientifique. Les rapports des évaluateurs de la première étape sont disponibles sur RechercheNet.

Veuillez consulter l'*Avis de décision* pour plus d'information sur le rang de votre demande.

Pour plus d'information sur le processus d'évaluation par les pairs du Programme de subventions Projet, veuillez consulter le *Guide d'évaluation par les pairs – Projet* (http://www.cihr-irsc.gc.ca/f/49564.html).
